# Risk factors for brain metastases after prophylactic cranial irradiation in small cell lung cancer

**DOI:** 10.1038/srep42743

**Published:** 2017-02-16

**Authors:** Haiyan Zeng, Peng Xie, Xue Meng, Shuanghu Yuan, Xindong Sun, Wanlong Li, Bingjie Fan, Xiaolin Li, Jinming Yu

**Affiliations:** 1 School of Medicine and Life Sciences, University of Jinan-Shandong Academy of Medical Sciences, Jinan, Shandong 250022, China; 2 Department of Radiation Oncology, Shandong Cancer Hospital Affiliated to Shandong University, Jinan 250117, Shandong, China; 3 Shandong Academy of Medical Sciences, Jinan 250022, Shandong, China

## Abstract

Despite administration of prophylactic cranial irradiation (PCI), some small cell lung cancer (SCLC) patients still suffer from brain metastases (BM) with unknown risk factors. We conducted this study to identify patients with higher BM risk after PCI and improve their outcome. The characteristics and survival of all the SCLC patients underwent PCI in our institute from 2003 to 2014 were analyzed. Kaplan-Meier method was applied to estimate BM free survival (BMFS) and overall survival (OS). Cox regression analyses were performed to explore risk factors for BM. A total of 175 patients with the median age of 55 years (range, 29–76) were eligible, among whom 36 (20.6%) developed BM with median follow-up of 42 months. Both univariate and multivariate analyses showed HART and TNM classification (*p* < 0.05) were associated with BM. Two-stage system was not related with BMFS or OS (*p* > 0.05). Stage IIIB-IV and HART were independent risk factors for BM after PCI in SCLC. TNM classification was more valuable on prognosis than two-stage system. Further large-scale studies are needed to confirm our findings.

Lung cancer is still the most frequently diagnosed malignant carcinoma and the leading cause of cancer death worldwide^1^, among which about 13–20% is small cell lung cancer (SCLC)^2^. Although being outstanding with high prevalence of brain metastases (BM), great progress has been made in SCLC during the past decades. One landmark achievement was applying prophylactic cranial irradiation (PCI) to limited-stage disease (LD) patients with complete response (CR) to initial therapy^3^ and another famous advance was delivering PCI to extensive-stage disease (ED) patients with response to initial therapy^4^. Since PCI not only produces a reduction in BM incidence but also provides an improvement in survival^5^, published guidelines always recommend PCI for both LD and ED patients who have a good response to initial treatment^6^,^7^. Yet, some patients still cannot avoid developing BM despite they have adopted PCI^4^,^8^,^9^,^10^. It remains unestablished that what factors cause some patients develop BM after PCI. Therefore, we conducted this research to find high risks for BM after PCI in SCLC and to offer some bases for further reducing BM rate and improving survival.

## Methods and Materials

We reviewed SCLC patients who underwent PCI in our institute from 2003 to 2014. Staging was done by bronchoscopy with biopsy, thoracic and abdominal computerized tomography (CT) with contrast medium, cranial magnetic resonance imaging (MRI) or CT with contrast medium and radionuclide bone scanning, or positron emission tomography/computerized tomography (PET/CT). Patients were staged according to TNM classification of the seventh edition American Joint Committee on Cancer (AJCC 7^th^ edition)^11^ and two-stage system based on version 1. 2016 of National Comprehensive Cancer Network Guidelines for SCLC (NCCN 2016)^6^.

Either HART or once-daily radiotherapy (QDRT) was adopted for thoracic radiotherapy according to different radiotherapy departments’ preferences (in some departments, all patients received HART; while in other departments, all patients received QDRT). Sequential chemoradiotherapy (SCRT) was delivered after 2–4 cycles of etopside-platinum chemotherapy if patients could not tolerant concurrent chemoradiotherapy (CCRT). A second contrast-enhanced cranial imaging was performed to patients prior to PCI to exclude immediate BM^12^. PCI to the whole brain was delivered mostly within two months after the end of chemoradiotherapy with the use of two opposed lateral fields to patients who have a response to initial therapy. The most commonly used schedule for HART, QDRT and PCI was 45 Gy/30 f over 3 weeks, 60 Gy/30 f over 6 weeks and 25 Gy/10 f over 2 weeks, respectively. Response to chemoradiotherapy was assessed with Response Evaluation Criteria in Solid Tumors (RECIST) 1.1 criteria^13^.

The primary endpoint was BM-free survival (BMFS) and the secondary endpoint was overall survival (OS), which were defined as time from pathological diagnosis to radiology confirmed BM and death or censorship at December, 2015, respectively. SPSS 18.0 was used to perform statistical analyses. Survival were estimated by Kaplan-Meier method and compared by means of log-rank tests. Cox regression analyses were performed to determine risk factors for BM. All tests were 2-sided and a *p* value less than 0.05 was considered to be statistically significant.

## Results

One hundred seventy-five patients underwent PCI were eligible, of whom 20 (11.4%) were with ED and 18 (11.5%) were with stage IV (Table 1, Supplementary Table 1). Due to the small sample size (n < 10) of stage IA, IB and IIB, TNM classification was analyzed by combining stage IA-IIIA and stage IIIB-IV. Before PCI, 62% of patients performed contrast-enhanced cranial CT, the other 38% performed contrast-enhanced cranial MRI.

**Figure 1:**
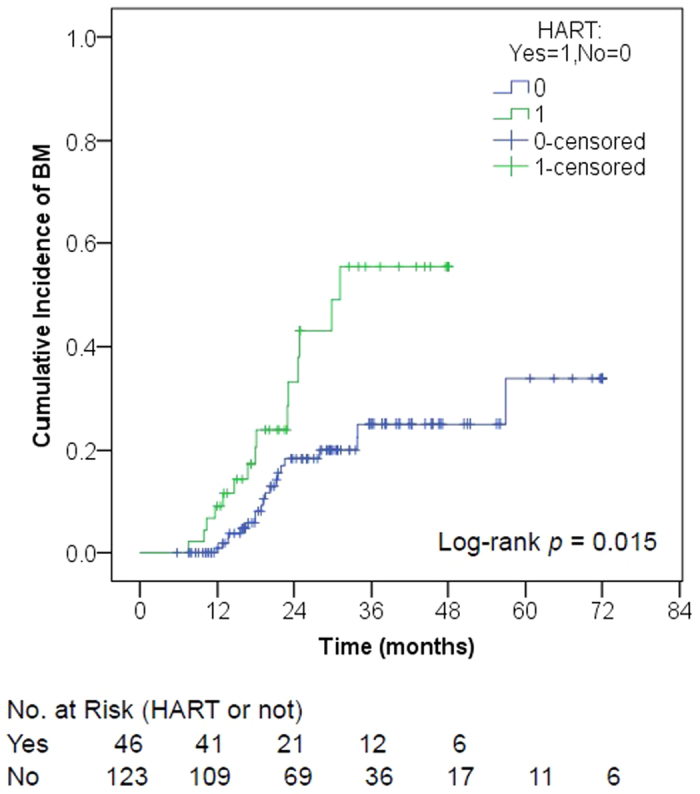
Cumulative incidence of brain metastases by HART. Brain metastases incidence was significantly higher in patients with thoracic hyperfractionated accelerated radiotherapy (HART) compared to those with once-daily radiotherapy (QDRT) (*p* = 0.015).

Among the 175 patients, 36 (20.6%) developed BM at a median follow-up time of 42.1 months (range, 7.4–119.4). Fourteen (38.9%) patients were symptomatic. The 5 year BMFS and OS rate was 69% and 48%, respectively. Univariate analyses showed that HART (*p* = 0.023, HR = 2.171, 95% CI 1.111–4.243) and TNM classification (*p* = 0.009, HR = 2.525, 95% CI 1.259–5.064) were significant variables associated with BM but two-stage system was not (*p* = 0.273) (Table 1, Fig. 1). The TNM × HART interaction was non-significant (*p* = 0.139, HR = 1.696, 95% CI 0.842–3.416). In the multivariate hazard model including TNM classification, HART (*p* = 0.014, HR = 2.748, 95%CI 1.227–6.157) was an independent high risk for BM; TNM classification (*p* = 0.073, HR = 2.119, 95%CI 0.932–4.821) tended to be an independent risk factor for BM (Table 1). While in the multivariate hazard model including two-stage system, HART (*p* = 0.026, HR = 2.448, 95% CI 1.116–5.372) was still an independent high risk for BM, but two-stage system (*p* = 0.280) was not (Table 1).

In addition, HART increased BM risk for patients with early stage SCLC no matter by stratum of TNM classification (Supplementary Table 2) or two-stage system (Supplementary Table 3) but had no significant influence on OS (median, 53.7 months vs. 46.9 months for HART vs. QDRT group, *p* = 0.570) (Supplementary Fig. 1). TNM classification was associated with OS (*p* = 0.010, HR = 2.002, 95% CI 1.180–3.395), but two-stage system was not (*p* = 0.728). Contrast-enhanced cranial CT or MRI prior to PCI was not related to either BMFS (*p* = 0.362) or OS (*p* = 0.239).

## Discussion

As an aggressive tumor, SCLC is outstanding with high prevalence of BM. PCI is an effective management to decrease BM rate and improve outcome. Unfortunately, some patients still develop BM despite they have administered PCI. To identify patients who are at-risk for developing BM after PCI is beneficial to help clinicians modifying the combined modality schedule for SCLC. In this study, we noted that thoracic HART and stage IIIB-IV were independent risk factors.

In line with the results of ECOG 2597 study on non-small cell lung cancer (NSCLC)^14^, HART failed to show survival benefit but did demonstrate a significant trend towards an increased risk of BM after PCI compared with HART in our research. The North Central Cancer Treatment Group (NCCTG) initiated a phase III study involved in 262 LD-SCLC patients to test the question of whether HART could improve upon the outcome of QDRT^15^. They found no differences between these two regimens with respect to local-only progression rates, overall progression rates or OS. Many other studies identified no survival benefit in the HART group^16^,^17^,^18^, either. Of course, some studies did show different results. ECOG initiated another phase III randomized trial and showed that HART significantly improved the median survival with higher incidence of grade 3 esophagitis^19^. However, both cohorts received a total of 45 Gy in this trial during 1989–1992. Studies reported later have shown that higher doses of 60–70 Gy should be used for QDRT^20^,^21^. The relatively lower dose of 45 Gy might account for the shorter OS in the QDRT group.

Overall, HART did not shown an obviously significant advantage over survival. On the contrary, HART was associated with higher BM incidence, especially for patients with earlier stage disease, because they lived longer. Since the TNM-by-HART interaction was non-significant, which was also confirmed in the multivariate analyses, HART was an independent risk for BM after PCI in SCLC. The underlying mechanism needs further exploration. One possible explanation is that HART shortens the irradiation interval, increases injuries of the normal tissue, including vessels and spine, which consist of the blood-spinal cord barrier (BSCB). The tumor cells can infiltrate across the impaired BSCB easier and generate BM with cerebrospinal fluid circulation months later. The international phase III study RTOG 0538 is ongoing and will further interpret the effects of HART in addition to PCI on BM and survival^22^.

Although it is recommended that two-stage system should be replaced by TNM classification^7^, the former is still extensively applied in clinical practice because of its simplicity. We analyzed both of them to compare their predictive power. Similar to previous reports, TNM classification was associated with both BMFS and OS in our study. But two-stage system showed insignificant association with either BMFS or OS. This’s due to the exclusion of most patients with ED since they were lack of PCI. Comparing these two staging schemes based on the same population, it’s obvious that TNM classification allowed for more precise prognostic assessments than two-stage system, mainly because the former integrates tumor, lymph nodes and metastases descriptors^11^, whereas the latter is primarily based on whether the tumor or nodal volume can be encompassed in a tolerable radiation plan.

On the other hand, our result that compared to those with LD-SCLC, patients with ED-SCLC did not show higher BM incidence after PCI supported the conclusion of the EORTC trial conducted by Slotman *et al*. that PCI should be part of standard care for all patients with ED-SCLC who have a response to initial therapy^4^. But some concerns did exist in this trial. The cranial imaging to confirm the absence of BM was not part of the staging and even follow up procedures. Contrast-enhanced CT or MRI was only performed to patients developed symptoms of BM such as headache and vomiting. The results might be biased by the neglected patients with asymptomatic BM before enrollment and after treatment. In our study, only 38.9% patients were diagnosed BM with symptoms, the other 60.1% were revealed asymptomatic BM by cranial imaging. Hochstenbag *et al*. also found a 15% asymptomatic BM rate detected by contrast-enhanced MRI of the brain at the initial diagnosis in patients with SCLC^23^. Manapov *et al*. revealed that the prevalence of asymptomatic BM immediately before PCI detected by a second contrast-enhanced MRI was as high as 32.5%, too^12^.

The phase III trial conducted by Seto *et al*. overcame the above shortcomings by screening patients with cranial MRI prior to enrollment and evaluating time to BM every 3 months by imaging^10^. It showed that PCI decreased BM risk but shortened OS for patients with ED-SCLC. Of note is that 330 patients were needed for this trial, which was terminated because the interim analysis including 163 patients found negative results. It’s unknown that whether the results would be different if a larger sample with 167 more patients being enrolled was analyzed.

In our study, cranial imaging with contrast medium was also part of staging before therapy. Either contrast-enhanced CT or MRI of the brain was repeated prior to PCI based on patients’ economic status. Although contrast-enhanced cranial CT has long been proved to have limited value in neurologically asymptomatic patients^24^,^25^, we did not find that patients performed CT before PCI was at higher risk to develop BM later compared to those performed MRI. This’s mainly because contrast-enhanced CT is sensitive and accurate to detect most BM^24^. As for the left population with occult BM that could be detected by MRI but could not be detected by CT, the micro-metastases could be eliminated by the following PCI^26^.

Additionally, we found the OS rate was higher than previous reports^3^,^9^,^19^,^27^, partly because all the patients in our study had undergone PCI, which has a benefit in survival. This’s also in part due to the relatively better status in patients who are appropriate for delivering PCI. What’s more, the different definition of OS also accounts for the different results, since most studies defined OS from randomization while ours from pathologic diagnosis.

In conclusion, our study demonstrated that HART and stage IIIB-IV were high risks for developing BM after PCI in patients with SCLC. The TNM classification was a better prognostic factor for both BM and survival than the two-stage system. Further large-scale investigations are needed to confirm our findings.

## Additional Information

**How to cite this article**: Zeng, H. *et al*. Risk factors for brain metastases after prophylactic cranial irradiation in small cell lung cancer. *Sci. Rep.* **7**, 42743; doi: 10.1038/srep42743 (2017).

**Publisher's note:** Springer Nature remains neutral with regard to jurisdictional claims in published maps and institutional affiliations.

## Supplementary Material

Supplementary Tables and Figures

## Figures and Tables

**Table 1:** Patients’ clinical features and BM risk analyses.

	Total	BM	BM rate	Univariate	Multivariate				
			3-year	*p*	*p*	HR	95% CI		
Gender									
Male	129	27	27	0.760	0.688				
Female	46	9	27		0.960†				
Age (years)									
<60	117	24	27	0.850	0.739				
≥60	58	12	27		0.439†				
Smoking history									
Yes	106	12	25	0.572	0.577				
No	67	22	26		0.700†				
NA	2	2							
TNM-classification (AJCC 7^th^ edition)									
IA-IIIA	83	13	19	0.009	0.073	2.119	0.932–4.821		
IIIB-IV	74	21	41						
NA‡	18	2							
Two-stage system (NCCN 2016)									
LD	155	30	26	0.273					
ED	20	6	35		0.280†				
HART									
Yes	46	15	43	0.023	0.014	2.748	1.227–6.157		
No	123	20	21		0.026†	2.448†	1.116–5.372†		
NA	6	1							
Response									
CR	65	16	29	0.842	0.409				
PR/SD	98	19	27		0.433†				
NA	12	1							
CCRT									
Yes	75	11	19	0.163	0.598				
No	98	25	33		0.365†				
NA	2	0							
Chemotherapy cycles									
≤6	159	33	27	0.960	0.167				
>6	14	3	27		0.298†				
NA	2	0							
Brain imaging prior to PCI									
CT				0.365	0.653				
MRI					0.482†				
NA									

*Abbreviations*: BM = brain metastases; HR = hazard ratio; CI = confidence interval; NA = non-applicable; AJCC = American Joint Committee on Cancer; NCCN = National Comprehensive Cancer Network; LD = limited-stage disease; ED = extensive-stage disease; HART = hyperfractionated accelerated radiation therapy; CR = complete response; PR = partial response, SD = stable disease; CCRT = concurrent chemoradiotherapy; CT = Computerized Tomography; MRI = Magnetic Resonance Imaging.

^†^Please read the results separately. The values with “†” represent results of the multivariate Cox regression analysis using two-stage system instead of TNM-classification.

^‡^Most patients were clinically staged using two-stage system and the TNM classification was retrospectively staged based on CT scan, which were not available for some patients so their TNM were NA.

## References

[b1] Torre L. A. . Global cancer statistics, 2012. CA: a cancer journal for clinicians 65, 87–108 (2015). 25651787 10.3322/caac.21262

[b2] Amini A., Byers L. A., Welsh J. W. & Komaki R. U. Progress in the management of limited-stage small cell lung cancer. Cance 120, 790–798 (2014). 10.1002/cncr.28505 PMC3947683 24327434

[b3] Auperin A. . Prophylactic cranial irradiation for patients with small-cell lung cancer in complete remission. Prophylactic Cranial Irradiation Overview Collaborative Group. The New England journal of medicine 341, 476–484 (1999). 10441603 10.1056/NEJM199908123410703

[b4] Slotman B. . Prophylactic cranial irradiation in extensive small-cell lung cancer. The New England journal of medicine 357, 664–672 (2007). 17699816 10.1056/NEJMoa071780

[b5] Schild S. E. . Prophylactic cranial irradiation in small-cell lung cancer: findings from a North Central Cancer Treatment Group Pooled Analysis. Annals of oncology: official journal of the European Society for Medical Oncology 23, 2919–2924, doi: 10.1093/annonc/mds123 (2012). 22782333 PMC3577038

[b6] Gregory P. & Kalemkerian B. W. L. Jr. *National Comprehensive Cancer Network (NCCN): NCCN Guidelines: Small Cell Lung Cancer, Version 1.2016.*, http://www.nccn.org/professionals/physician-gls/pdf/sclc.pdf (2016).

[b7] Fruh M. . Small-cell lung cancer (SCLC): ESMO Clinical Practice Guidelines for diagnosis, treatment and follow-up. Annals of oncology: official journal of the European Society for Medical Oncology 24, 27 (2013). 10.1093/annonc/mdt178 23813929

[b8] Naidoo J., Kehoe M., Sasiadek W., Hacking D. & Calvert P. Prophylactic cranial irradiation in small cell lung cancer: a single institution experience. Ir J Med Sci 183, 129–132, doi: 10.1007/s11845-013-0977-z (2014). 23760883

[b9] Slotman B. J. . Use of thoracic radiotherapy for extensive stage small-cell lung cancer: a phase 3 randomised controlled trial. The Lancet 385, 36–42, doi: 10.1016/s0140-6736(14)61085-0 (2015). 25230595

[b10] Takashi Seto T. T. *et al. Prophylactic cranial irradiation (PCI) has a detrimental effect on the overall survival (OS) of patients (pts) with extensive disease small cell lung cancer (ED-SCLC): Results of a Japanese randomized phase III trial*. http://meetinglibrary.asco.org/content/129034-144(2014).

[b11] Goldstraw P. . The IASLC Lung Cancer Staging Project: proposals for the revision of the TNM stage groupings in the forthcoming (seventh) edition of the TNM Classification of malignant tumours. Journal of thoracic oncology: official publication of the International Association for the Study of Lung Cancer 2, 706–714 (2007). 10.1097/JTO.0b013e31812f3c1a 17762336

[b12] Manapov F., Klautke G. & Fietkau R. Prevalence of brain metastases immediately before prophylactic cranial irradiation in limited disease small cell lung cancer patients with complete remission to chemoradiotherapy: a single institution experience. Journal of thoracic oncology: official publication of the International Association for the Study of Lung Cancer 3, 652–655 (2008). 10.1097/JTO.0b013e3181757a76 18520807

[b13] Eisenhauer E. A. . New response evaluation criteria in solid tumours: revised RECIST guideline (version 1.1). European journal of cancer (Oxford, England: 1990) 45, 228–247 (2009). 10.1016/j.ejca.2008.10.026 19097774

[b14] Belani C. P. . Phase III study of the Eastern Cooperative Oncology Group (ECOG 2597): induction chemotherapy followed by either standard thoracic radiotherapy or hyperfractionated accelerated radiotherapy for patients with unresectable stage IIIA and B non-small-cell lung cancer. Journal of clinical oncology: official journal of the American Society of Clinical Oncology 23, 3760–3767 (2005). 15837967 10.1200/JCO.2005.09.108

[b15] Bonner J. A. . Phase III comparison of twice-daily split-course irradiation versus once-daily irradiation for patients with limited stage small-cell lung carcinoma. Journal of clinical oncology: official journal of the American Society of Clinical Oncology 17, 2681–2691 (1999). 10561342 10.1200/JCO.1999.17.9.2681

[b16] Schild S. E. . Long-term results of a phase III trial comparing once-daily radiotherapy with twice-daily radiotherapy in limited-stage small-cell lung cancer. International journal of radiation oncology, biology, physics 59, 943–951 (2004). 10.1016/j.ijrobp.2004.01.055 15234027

[b17] Komaki R. . Phase II study of accelerated high-dose radiotherapy with concurrent chemotherapy for patients with limited small-cell lung cancer: Radiation Therapy Oncology Group protocol 0239. International journal of radiation oncology, biology, physics 83, 5 (2012). 10.1016/j.ijrobp.2012.01.075 PMC3377848 22560543

[b18] Gazula A., Baldini E. H., Chen A. & Kozono D. Comparison of once and twice daily radiotherapy for limited stage small-cell lung cancer. Lung 192, 151–158 (2014). 24162870 10.1007/s00408-013-9518-9

[b19] Turrisi A. T. 3rd . Twice-daily compared with once-daily thoracic radiotherapy in limited small-cell lung cancer treated concurrently with cisplatin and etoposide. The New England journal of medicine 340, 265–271 (1999). 9920950 10.1056/NEJM199901283400403

[b20] Miller K. L. . Routine use of approximately 60 Gy once-daily thoracic irradiation for patients with limited-stage small-cell lung cancer. International journal of radiation oncology, biology, physics 56, 355–359 (2003). 10.1016/s0360-3016(02)04493-0 12738309

[b21] Bogart J. A. . 70 Gy thoracic radiotherapy is feasible concurrent with chemotherapy for limited-stage small-cell lung cancer: analysis of Cancer and Leukemia Group B study 39808. International journal of radiation oncology, biology, physics 59, 460–468, doi: 10.1016/j.ijrobp.2003.10.021 (2004). 15145163

[b22] Bogart J. A. Phase III Comparison of Thoracic Radiotherapy Regimens in Patients With Limited Small Cell Lung Cancer Also Receiving Cisplatin and Etoposide, https://www.clinicaltrials.gov/ct2/show/study/NCT00632853 (2016).

[b23] Hochstenbag M. M., Twijnstra A., Wilmink J. T., Wouters E. F. & ten Velde G. P. Asymptomatic brain metastases (BM) in small cell lung cancer (SCLC): MR-imaging is useful at initial diagnosis. Journal of neuro-oncology 48, 243–248 (2000). 11100822 10.1023/a:1006427407281

[b24] Johnson D. H., Windham W. W., Allen J. H. & Greco F. A. Limited value of CT brain scans in the staging of small cell lung cancer. AJR. American journal of roentgenology 140, 37–40, doi: 10.2214/ajr.140.1.37 (1983). 6295121

[b25] Habets J. M., van Oosterhout A. G., ten Velde G. P., Wilmink J. T. & Twijnstra A. Diagnostic value of CT in the detection of brain metastasis in small cell lung cancer patients. Journal belge de radiologie 75, 179–181 (1992). 1328148

[b26] Hu X. & Chen M. Prophylactic cranial irradiation for limited-stage small cell lung cancer: controversies and advances. Zhongguo Fei Ai Za Zhi 16, 373–377, doi: 10.3779/j.issn.1009-3419.2013.07.08 (2013). 23866669 PMC6000652

[b27] Corso C. D. . Role of Chemoradiotherapy in Elderly Patients With Limited-Stage Small-Cell Lung Cancer. Journal of clinical oncology: official journal of the American Society of Clinical Oncology 33, 4240–4246 (2015). 26481366 10.1200/JCO.2015.62.4270 PMC4678178

