# Supplementary material for: Risk factors for brain metastases after prophylactic cranial irradiation in small cell lung cancer
Source: Sci Rep. 2017 Feb 16;7:42743. doi: 10.1038/srep42743 (PMC5311871; doi:10.1038/srep42743)
Supplement: Supplementary Tables and Figures [file srep42743-s1.pdf]

Risk factors for brain metastases after prophylactic cranial irradiation in small cell lung cancer

Haiyan Zeng[1,2#], Peng Xie[3#], Xue Meng[2,4], Shuanghu Yuan[2,4], Xindong Sun[2,4], Wanlong Li[2,4], Bingjie Fan[2,4],Xiaolin Li[2,4], Jinming Yu[2,4*].

Supplementary table 1 Patients' characteristics

	No.(median)	Constituent ratio (%)(range)
Gender		
Male	129	73.7
Female	46	26.3
Age (years)	55	29-76
PS		
0	10	5.7
1	162	92.6
2	3	1.7
Smoking history		
Yes	106	61.3
No	67	38.7
NA	2	
Two-stage system (NCCN 2016)		
LD	155	88.6
ED	20	11.4
TNM classification (AJCC 7th edition)		
IA	3	1.9
IB	4	2.5
IIA	10	6.4
IIB	1	6.0
IIIA	65	41.4
IIIB	56	35.7
IV	18	11.5
NA[†]	18	
Brain imaging prior to PCI[††]		
CT	93	62.0
MRI	57	38.0
NA	25	

Abbreviations: PS = performance status; NA = non-applicable; NCCN = National Comprehensive Cancer Network; LD = limited-stage disease; ED = extensive-stage disease; AJCC = American Joint Committee on Cancer; CT = Computerized Tomography; MRI = Magnetic Resonance Imaging.

Note: [†] Most patients were clinically staged using two-stage system and the TNM classification was retrospectively staged based on CT scan, which were not available for some patients so their TNM were NA. [††]Contrast-enhanced brain imaging.

Supplementary table 2

Univariate Cox analysis for BM risk by stratum of TNM classification (AJCC 7th edition)

	IA-IIIA (N=83)			IIIB-IV (N=74)		
	p	HR	95%CI	*p*	HR	95%CI
Sex	0.936	1.049	0.323 - 3.412	0.369	0.627	0.227 - 1.736
Age	0.736	0.801	0.220 - 2.912	0.945	0.970	0.401 - 2.342
Smoking	0.547	0.705	0.227 - 2.195	0.657	0.802	0.304 - 2.118
HART	0.013	4.011	1.347 - 11.946	0.512	1.366	0.538 - 3.471
Response	0.430	0.644	0.216 - 1.920	0.593	1.289	0.508 - 3.270
CCRT	0.088	0.325	0.089 - 1.182	0.786	0.882	0.356 - 2.187
Chemotherapy cycles	0.225	2.224	0.612 - 8.083	0.550	0.045	0.000 - 1160.422
Brain imaging prior to PCI	0.477	0.617	0.164 - 2.331	0.935	0.958	0.339 - 2.705

Abbreviations: BM = brain metastases; HR = hazard ratio; CI = confidence interval; AJCC = American Joint Committee on Cancer; HART = hyperfractionated accelerated radiotherapy; CCRT = concurrent chemoradiotherapy; PCI = prophylactic cranial irradiation.

Supplementary table 3 Univariate Cox analysis for BM risk by stratum of two-stage system (NCCN 2016)

	LD (N=155)			ED (N=20)		
	p	HR	95%CI	*p*	HR	95%CI
Sex	0.840	1.087	0.484 - 2.444	0.866	0.826	0.089 -7.629
Age	0.942	1.031	0.458 - 2.318	0.281	0.368	0.060 - 2.268
Smoking	0.933	0.968	0.458 - 2.049	0.457	30.158	0.004-239789.249
HART	0.036	2.164	1.051 - 4.455	0.231	3.490	0.452 - 26.950
Response	0.984	1.008	0.489 - 2.078	0.751	0.727	0.102 - 5.185
CCRT	0.165	0.575	0.263 - 1.256	0.970	0.966	0.161 - 5.814
Chemotherapy cycles	0.744	1.220	0.370 - 4.022	0.766	0.045	0.000-3195830.81
Brain imaging prior to PCI	0.516	0.743	0.303 - 1.823	0.151	0.184	0.018 - 1.858

Abbreviations: BM = brain metastases; HR = hazard ratio; CI = confidence interval; NCCN = National Comprehensive Cancer Network; LD = limited-stage disease; ED = extensive-stage disease; HART = hyperfractionated accelerated radiotherapy; CCRT = concurrent chemoradiotherapy; PCI = prophylactic cranial irradiation.

Supplementary figure 1

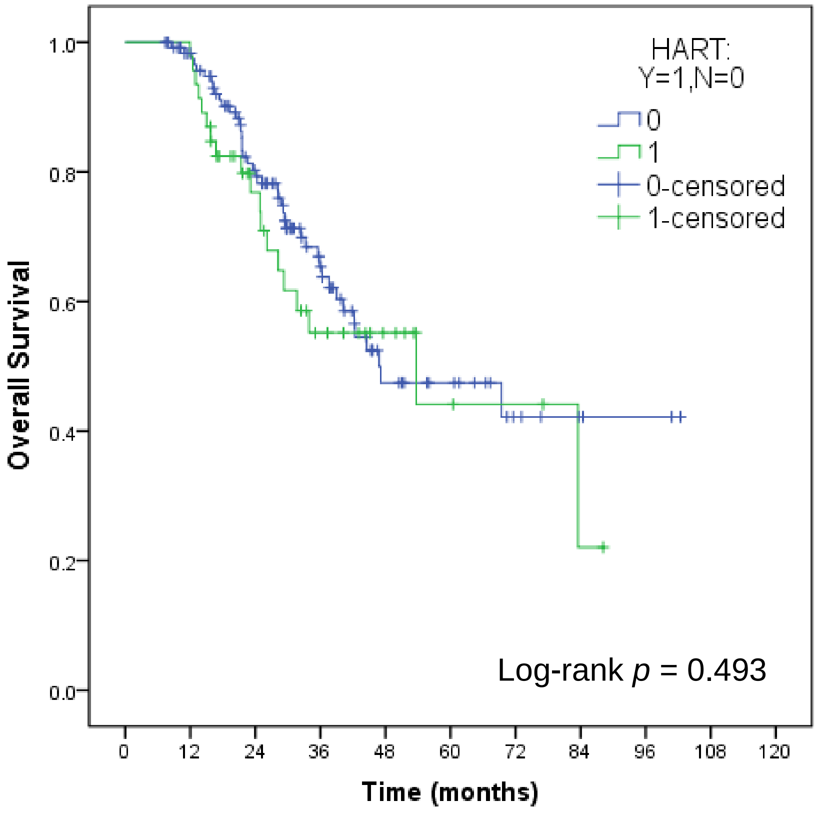

No. at Risk (HART or not)

Yes	46	45	26	15	9	4	3	1	
No	123	112	79	42	19	14	6	3	2

Supplementary legends

Supplementary figure 1. Kaplan-Meier estimates of overall survival by HART. Overall survival (BM) was not significantly different in patients with thoracic hyperfractionated accelerated radiotherapy (HART) compared to those with once-daily radiotherapy (QDRT) (p = 0.493).
